# Reciprocal Effects Between Life Satisfaction and Sleep Duration in Chinese Older Adults

**DOI:** 10.1093/geroni/igaa057.737

**Published:** 2020-12-16

**Authors:** Qian Liu, Haimin Pan, Yaolin Pei

**Affiliations:** 1 Hunan Normal University, Changsha, China; 2 Xiamen University, Xiamen, China; 3 New York University, New York, New York, United States

## Abstract

Sleep problems are prevalent among older adults. It is evident that sleep duration, as an important indicator of sleep quality, is closely associated with life satisfaction. However, it remains unclear as to whether sleep duration influences life satisfaction, or whether lower levels of life satisfaction increase the likelihood of sleep duration decline. This study examined the directional relationship between life satisfaction and sleep duration among Chinese older adults. Data were derived from the China Health and Retirement Longitudinal Study (2011, 2013, 2015 waves; age≥60 year; n = 5689). A cross-lagged panel model (path analysis) with three time points was used to jointly examine the longitudinal reciprocal effects between life satisfaction and sleep duration. This model achieved acceptable indices of goodness of fit. The results revealed that sleep duration were positively associated with life satisfaction at all time points (2011 to 2013: B=.06,SE=.01,p<.0001; 2013 to 2015: B=.05,SE=.01,p<.0001), and life satisfaction also positively predicted sleep duration across timepoints (2011 to 2013: B=.07,SE=.01,p<.0001; 2013 to 2015: B=.03,SE=.01,p<.05).These associations remained unchanged when taking demographics, and noontime napping into account. The findings indicate that the relationship between life satisfaction and sleep duration is bidirectional. Sleep duration may present as a mechanism for the relationship between life satisfaction and health, and suggests that effective treatment of sleep duration may improve life satisfaction.

